# AKR2A is involved in the flowering process of *Arabidopsis thaliana*

**DOI:** 10.1080/15592324.2022.2100685

**Published:** 2022-07-22

**Authors:** Qian Tang, Ya-Nan Zhao, Shan Luo, Shan Lu

**Affiliations:** State Key Laboratory of Pharmaceutical Biotechnology, School of Life Sciences, Nanjing University, Nanjing, China

**Keywords:** *Arabidopsis thaliana*, ANKYRIN REPEAT-CONTAINING PROTEIN 2A (AKR2A), FLOWERING LOCUS T (FT), flowering time

## Abstract

Flowering at an appropriate time is crucial for plant development and reproduction. In *Arabidopsis*, the flowering process is managed by a regulatory network composed of at least 6 independent pathways. As a core protein in flowering regulation, FLOWERING LOCUS T (FT) participates in almost all these pathways. ANKYRIN REPEAT-CONTAINING PROTEIN 2A (AKR2A) was initially discovered as a 14-3-3-interacting protein. It was then found to be involved in the transportation of chloroplast outer membrane proteins and the resistance to low-temperature stress. Here, we identified an *akr2a* null mutant with a delayed flowering phenotype. Through the quantitative real-time PCR (qRT-PCR) and bimolecular fluorescence complementation (BiFC) assays, we demonstrated that AKR2A modulates the flowering process through its interaction with FT.

## Introduction

Flowering is not only the transition from vegetative growth to reproductive growth but also the most significant change in the plant growth cycle.^1^ In the process of plant development, the flowering time is determined by a complex and elaborate molecular mechanism.^2^ Up to now, more than 300 genes have been identified in the flowering process of *Arabidopsis thaliana*.^3^ Six pathways co-regulate flower induction, *i.e*., the photoperiod pathway, vernalization pathway, ambient temperature pathway, gibberellin pathway, autonomous pathway, and age pathway.^4^ These pathways are independent and cooperate to form a complex and precise regulatory network.

FLOWERING LOCUS T (FT) is one of the most critical genes in the flowering regulation pathway.^5–7^ FT encodes a florigen protein that moves from leaves to plant growth points and activates the expression of downstream genes, thus promoting the transformation from vegetative growth to reproductive growth.^8,9^ Usually, CONSTANS (CO), induced by the long-day signal, binds to the promoter of *FT*, and activates its downstream genes.^10^ On the other hand, the transcription inhibitor Flowing LOCUS C (FLC) inhibits the expression of *FT* and negatively regulates *Arabidopsis* flowering.^11^

*Arabidopsis thaliana* ANKYRIN REPEAT-CONTAINING PROTEIN 2A (AKR2A) was recognized to function in disease resistance, antioxidation metabolism, and chloroplast outer membrane protein targeting.^12–15^ It has been reported to interact with peroxisomal membrane-bound ASCORBATE PEROXIDASE3 (APX3), 3-ketoacyl-CoA synthase 1 (KCS1), and chloroplast protein OEP7.^12–15^ Its mutants constructed using the antisense technique resulted in small necrotic areas in leaves accompanied by enhanced production of H_2_O_2_ under normal growth conditions.^16^ Under chilling temperature conditions, its point mutant T6 (Glu-to-Lys change at residue 150) seedlings were shorter and had smaller and curled rosette leaves compared with its wild-type (WT) parental line Columbia (Col) er105 (BM).^15^ A slightly delayed-flowering phenotype of the T6 mutant was also observed.^15^ However, these studies were not based on its null mutant, and some other functions of AKR2 might be missed.

In this study, we confirmed that the T-DNA insertion null mutant *akr2a* has a delayed-flowering phenotype, which might result from the protein-protein interaction between AKR2A and FT.

## Results and discussion

### AKR2A expression affects Arabidopsis flowering

**Figure 1.**
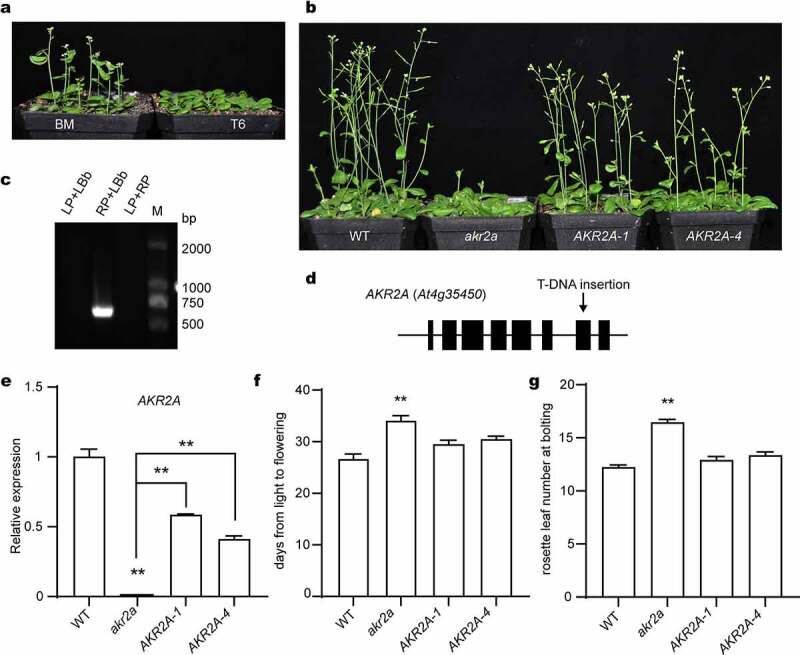
The silencing of *AKR2A* accounts for the delayed-flowering phenotype. (a) Flowering phenotype of the *AKR2A* point mutant T6 and its wild-type parent BM seedlings. (b) Flowering phenotype of the Col-0 WT, the T-DNA insertion *akr2a* mutant, and two complementation line (*AKR2A-2, AKR2A-4*) seedlings. For (a) and (b), seedlings were grown under a long-day (16 h/8 h light/dark) condition. Representative images were taken at 35 d after germination. (c) PCR confirmation of the T-DNA insertion *akr2a* mutant. (d) Scheme of the *AKR2A* gene structure. Black boxes indicate the exons. The arrow indicates the position of T-DNA insertion. (e) Relative expression of *AKR2A* in different lines at 28 d after germination. Transcript abundance was determined by qRT-PCR. *ACTIN2* served as a reference. Data are means ± SEM (*n* = 3, Student’s *t*-test, **P < .01). (f) and (g) The days from light to flowering (f) and the rosette leaf number at bolting (g) of different lines. Data are means ± SEM (*n* = 9, Student’s *t*-test, **P < .01).

To confirm the delayed-flowering phenotype of the *AKR2A* point-mutant T6, we acquired the T-DNA insertion line *akr2a* with a Col-0 background from ABRC. With the T-DNA insertion at the 7th exon, the transcript of *AKR2A* was not detected (Figure 1c, 1e). We then generated its homozygous complementation lines using its own promoter region to drive its full-length cDNA. All these lines were confirmed at the genomic and transcript levels (Figure 1c and 1e).

Under a 16 h/8 h light/dark long-day growth condition, the *akr2a* mutant seedlings flowered 7–8 d later than Col-0 WT, while the complementation seedlings flowered 4–5 d earlier than *akr2a* seedlings (Figure 1f). In the meantime, the rosette leaf number of *akr2a* was 4–6 more than Col-0 WT, but those of the two complementation lines were similar to the WT level (Figure 1g). Therefore, our results demonstrated the delayed flowering phenotype of the *AKR2A* null mutant.

### Deletion of *AKR2A* affects the expression of flowering-related genes

**Figure 2.**
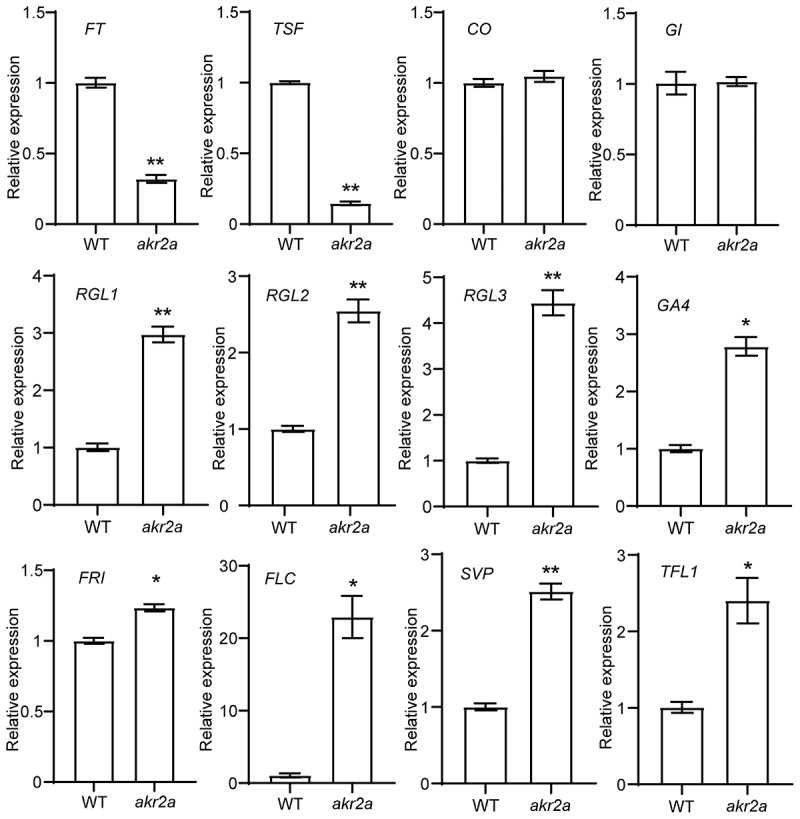
Transcript levels of flowering-related genes in *akr2a* mutant plants. Transcript abundances of flowering-related genes as indicated in *akr2a* mutant and the Col-0 WT seedlings at 28 d after germination were quantified by qRT-PCR. *ACTIN2* served as a reference. Data are means ± SEM (*n* = 3, Student’s *t*-test, *P < .05, **P < .01).

Flowering is the transition from vegetative to reproductive growth in response to developmental and environmental cues.^17^ In the photoperiodic pathway, *FT* and *TWIN SISTERS OF FT* (*TSF*) are activated by CO and positively regulate *Arabidopsis* flowering.^18,19^ At the earlier stages, GIGANTEA (GI) forms a complex on the *CO* promoter to regulate *CO* expression.^20^ In the *akr2a* mutant, transcript abundances of *FT* and *TSF* were significantly lower than that in WT, but thelevels of *CO* or *GI* were similar to their corresponding WT levels (Figure 2). DELLA proteins (RGL1, RGL2, RGL3) and GA4 were negative regulators of GA responses to flowering,^21–23^ and the transcript levels of all their coding genes significantly increased in *akr2a* (Figure 2). Previous studies have proved that FRIGIDA (FRI), FLOWERING LOCUS C (FLC), SHORT VEGETATIVE PHASE (SVP), and TERMINAL FLOWER 1 (TFL1) inhibit the flowering of *Arabidopsis* by the vernalization pathway, ambient temperature pathway, and autonomous pathway.^24–27^ We also tested the transcript levels of these genes, and found enhanced transcript abundances of these genes in *akr2a* seedlings (Figure 2). Taken together, the silencing of *AKR2A* affects the expression of a bouquet of flowering-related genes.

### *In vivo* interaction of AKR2A with FT

**Figure 3.**
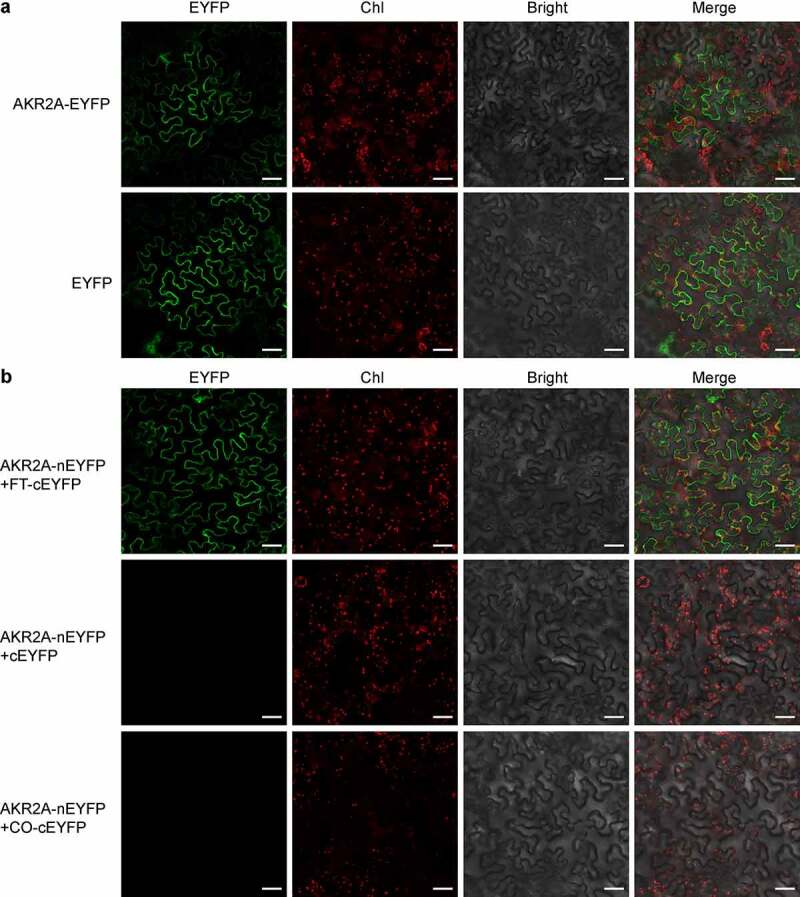
AKR2A interacts with FT in the cytosol. (a) Subcellular localization assay. Full-length AKR2A fused with EYFP or empty EYFP were transiently expressed in *Nicotiana benthamiana* mesophyll cells by infiltration. (b) BiFC detection. AKR2A was fused with the N-half of EYFP (nEYFP), and FT and CO were individually fused with the C-half of EYFP (cEYFP). Different combinations of fusion proteins as indicated were co-expressed in *N. benthamiana* mesophyll cells by infiltration. Images were collected 3 d after infiltration. Representative images under EYFP, chlorophyll (Chl), and bright field (Bright) channels and the merged signals are shown. Bar = 50 μm.

To further investigate how AKR2A regulates flowering time, we studied the subcellular localization of AKR2A. Full-length open-reading frame (ORF) of *AKR2A* was fused with enhanced yellow fluorescent protein (EYFP), and the fusion protein AKR2A-EYFP was transiently expressed in *Nicotiana benthamiana* mesophyll cells. As is shown in Figure 3a, AKR2A showed a cytosolic localization, resembling that of EYFP. We then tested whether AKR2A was able to interact with FT and/or CO, the two core components in flowering regulation. A BiFC assay was performed to test their *in planta* interactions. Our microscopic observation clearly revealed the positive interaction between AKR2A and FT in the cytosol, but not that between AKR2A and CO (Figure 3b).

In this work, by utilizing the T-DNA insertion null mutant, we demonstrated the function of AKR2A in regulating the flowering process in *A. thaliana*. By comparing the transcript levels of flowering-related genes between *akr2a* and WT plants, we found that the silencing of *AKR2A* resulted in the down-regulation of the positive control genes, such as *FT* and *TSF*. On the other hand, the negative control genes, such as *RGL, FRI*, and *FLC*, were upregulated in the *akr2a* mutant (Figure 2). Our BiFC observation indicated the protein-protein interaction between AKR2A and FT (Figure 3). Such an interaction might explain the delayed-flowering phenotype of *akr2a*. It is interesting that FT was also reported to interact with the 14-3-3 protein.^28^ The protein-protein interaction between AKR2A and FT in the cytosol, as observed in our BiFC assay, might affect the translocation of FT from the cytosol to the nucleus (Figure 3b). Further analysis of the relationships among AKR2A, 14-3-3 proteins, and AKR2A-interacting proteins will help to decipher the plethora of functions of AKR2A.^29^

## Materials and methods

### Plant materials and growth condition

The *akr2a* mutant lines (SALK_204151C, with the T-DNA insertion at the exon) and wild-type ecotype Columbia-0 (Col-0 WT) were obtained from the Arabidopsis Biological Resource Center (ABRC, Columbus, USA). Seeds of Col er105 (BM) and its *AKR2A* TILLING line (T6) were kindly provided by Dr. Guoxin Shen (Zhejiang Academy of Agricultural Sciences, Hangzhou, China).^13^

All seeds were stratified at 4°C in the dark for 3 d and then allowed to germinate on Murashige-Skoog (MS) plates containing 2% sucrose and 0.8% agar at 22°C under 120 µmol photons m^−2^ s^−1^ irradiance with a 16 h/8 h light/dark regime (as the long-day condition) and relative humidity of 60% in a growth chamber. Two-week-old seedlings were moved to grow in soil (a mixture of peat moss, vermiculite, and perlite at 1:1:1) under the same conditions.

To rescue the *akr2a* null mutant, we fused a 2-kb DNA fragment upstream of the translation initiation codon (ATG) of *AKR2A*, the full-length ORF of *AKR2A*, and a 700-bp DNA fragment downstream of the stop codon of *AKR2A* in a tandem array. The cassette was cloned into pCAMBIA1300 (CAMBIA, Canberra, Australia). The *akr2a* mutant seedlings were transformed with this construct using the *Agrobacterium*-mediated floral-dipping method.^30^ Transgenic plants were screened on MS plates with 40 μg mL^−1^ hygromycin B and further confirmed by qRT-PCR for the expression of corresponding transgenes.

### Molecular manipulation

Genomic DNA was extracted from *A. thaliana* leaves using the CTAB method.^31^ The presence of the T-DNA insertion was detected by PCR according to the SIGnAL iSect tool (http://signal.salk.edu/tdnaprimers.2.html).

For RNA isolation, leaves were ground in liquid nitrogen into a fine powder and extracted using the RNAiso reagent (TaKaRa, Shiga, Japan). For each sample, 1 μg of total RNA was used for synthesizing the first-strand cDNA using the HiScript III RT SuperMix for qPCR (+gDNA wiper) (Vazyme, Nanjing, China) following the manufacturer’s instruction. qRT-PCR was performed using ChamQ SYBR qPCR Master Mix (Vazyme) in a Thermal Cycler Dice Real Time System TP800 (TaKaRa) following the manufacturers’ instructions. The cycling program consisted of 95°C for 5 min, followed by 40 cycles of 95°C for 10s, and then 60°C for 15s. Transcript abundance of *ACTIN2* (*At3g18780*) was quantified as a reference. Relative expression levels were calculated by the comparative *C*_T_ method.^32^ All primer sequences used in this study are listed in Table S1.

### Subcellular localization and BiFC assays

For subcellular localization assay, the full-length ORF of *AKR2A* was cloned into the pCNHP-EYFP at the NcoI site as previously described.^33^ In this construct, the enhanced cauliflower mosaic virus (CaMV) 35S promoter, synthetic 5’ and 3’ untranslated regions of cowpea mosaic virus RNA-2, the coding region of *AKR2A*, enhanced yellow fluorescent protein (EYFP), and the Heat Shock Protein (HSP) terminator from *A. thaliana* were linked sequentially in the pCNHP-EYFP to express 35S:AKR2A-EYFP.^33^

For BiFC assay, the full-length ORF of *AKR2A* was cloned into pCNHP-nEYFP to generate 35S:AKR2A-nEYFP, and the full-length ORFs of *FT* and *CO* were individually cloned into pCNHP-cEYFP to generate 35S:FT-cEYFP and 35S:FT-cEYFP, respectively. The pCNHP-cEYFP and pCNHP-nEYFP vectors are almost the same as pCNHP-EYFP, except that they harbor the C- and N-halves of EYFP, respectively, instead of the intact EYFP of pCNHP-EYFP.

Each of the constructs was transformed into *Agrobacterium tumefaciens* strain GV3101 by electroporation.^34^ *Agrobacterium* cells were collected by centrifugation at 5000 *g* for 5 min and then resuspended in infiltration media (10 mM MES, 10 mM MgCl_2_, and 200 μM acetosyringone) to an OD_600_ of 1. Before infiltration on *Nicotiana benthamiana* leaves, the mixture was activated for about 3 h at room temperature. Finally, the transfected plants were allowed to grow for 3 d under a 16 h light/8 h dark light cycle.

Fluorescence signals were observed using a confocal laser scanning microscope (FluoView FV1000, Olympus, Tokyo, Japan). The excitation wavelength and the emission filters for EYFP were 515 nm and 530–560 nm, respectively. Chlorophyll autofluorescence was monitored using 543 nm excitation wavelengths and 680–720 nm detection windows.

### Statistical analysis

GraphPad Prism 9 (GraphPad Software, San Diego, CA, USA) was used for statistical analysis. We employed Student’s *t*-test to determine statistical significance. Differences were considered significant at *P* < .05 and *P* < .01 levels.
